# Global Impact of the COVID-19 Pandemic on Orthopedics and the Implications of Telemedicine: A Systematic Review of the Literature

**DOI:** 10.3390/jcm11112983

**Published:** 2022-05-25

**Authors:** Chia-Hao Hsu, Hsuan-Ti Huang, Chung-Hwan Chen, Yin-Chih Fu, Pei-Hsi Chou, Nin-Chieh Hsu

**Affiliations:** 1 Graduate Institute of Clinical Medicine, College of Medicine, Kaohsiung Medical University, No. 100, Shiquan 1st Rd., Sanmin District, Kaohsiung 80708, Taiwan; ecowarrior.tw@yahoo.com.tw (C.-H.H.); hwan@kmu.edu.tw (C.-H.C.); arthroscopy.pc@gmail.com (P.-H.C.); 2 Department of Orthopedics, Kaohsiung Municipal Ta-Tung Hospital, No. 68, Jhonghua 3rd Rd., Cianjin District, Kaohsiung 80145, Taiwan; microfu@ms.kmuh.org.tw; 3 Department of Orthopedics, Kaohsiung Medical University Hospital, No. 100, Tzyou 1st Rd., Sanmin District, Kaohsiung 80756, Taiwan; hthuang@kmu.edu.tw; 4 Department of Orthopedics, College of Medicine, Kaohsiung Medical University, No. 100, Shiquan 1st Rd., Sanmin District, Kaohsiung 80708, Taiwan; 5 Department of Internal Medicine, National Taiwan University Hospital, No. 7, Zhongshan S. Rd., Zhongzheng District, Taipei 10002, Taiwan

**Keywords:** COVID-19, pandemic, impact, orthopedics, telemedicine, virtual consultations, virtual teaching

## Abstract

This study aimed to systematically review the literature on the impact of the coronavirus disease (COVID-19) pandemic on the orthopedics field by focusing on multiple aspects, including orthopedic training and application, performance, work loading, change of practice, research work, and other psychological factors. Published articles were searched using the PubMed database. Articles were selected in accordance with the Preferred Reporting Items for Systematic Reviews and Meta-Analyses guidelines. Of 58 studies published between 1 January 2020 and 1 October 2021, 57 peer-reviewed original articles were included. Nearly 90% of students experienced an impact of the pandemic on application. The impact on training stemmed from redeployment rates of 20.9–23.1%. The rate of emergency or outpatient visits decreased from 18% to 58.6%. The rates of all surgeries or emergency surgeries decreased by 15.6–49.4%, while the rates of elective surgeries decreased by 43.5–100%. The rate of work loading ranged from 33% to 66%. Approximately 50–100% of surgeons had a change of practice. A total of 40.5% of orthopedic surgeons experienced mild psychological pressure. Approximately 64% had stopped research participant recruitment. Most of the included studies were conducted in Europe, followed by Asia and North America. It is suggested orthopedic surgeons prepare more sufficient, flexible, and reservable staffing measures, proper preventive strategies and surgical scheduling algorithms, and set up dedicated venues and equipment for routine telemedicine with staff training for virtual teaching or consultations in case of future impacts on orthopedics.

## 1. Introduction

Coronavirus disease (COVID-19) cases were first reported in Wuhan, China in December 2019 and the World Health Organization declared COVID-19 as a worldwide pandemic on 12 March 2020 [1]. Hundreds of millions of cases have been diagnosed, and the number continues to rise, not to mention millions of patients who have died from the disease, severely affecting the global economy. The global orthopedic field was inevitably impacted under this pandemic.

The COVID-19 related lockdowns or restrictions have dramatically changed the daily lives of people around the world. It had a major impact on the healthcare system and forced the orthopedic field to execute fundamental changes. To maximize the capacity to treat massive numbers of COVID-19 patients, hospitals have been forced to redeploy their employees. The pandemic has widely affected the field of orthopedics. Related preventive strategies have created many restrictions, such as diverting employees, postponing elective surgeries, suspending some outpatient clinics, stopping any training or teaching activities, and canceling non-urgent referrals or consultations to minimize exposure and clustering.

This systematic review aimed to obtain a comprehensive overview of the impact of the COVID-19 pandemic on orthopedics by analyzing previously published results from countries around the world, especially focusing on multiple aspects, including orthopedic training and application, performance, work loading, change of practice, research, and other psychological factors.

## 2. Materials and Methods

An extensive literature search was conducted for articles published from 1 January 2020 to 1 October 2021 to collect all specific publications since the outbreak of COVID-19. The PubMed database was used as the primary search database. However, if we included all orthopedic subspecialties, more than 1000 publications would be searched; thus, we restricted the search strategies to articles with titles that contained the following terms: ‘orthopedic’ or ‘orthopaedic’. We used the following search items: ‘impact’ AND ‘COVID-19’ AND ‘orthopedic’ plus ‘impact’ AND ‘COVID-19’ AND ‘orthopaedic’ in the title. The review was conducted in accordance with the Preferred Reporting Items for Systematic Reviews and Meta-Analyses (PRISMA) guidelines [2]. Based on the fact that the pandemic began just about 2 years ago, there may be a lack of prospective studies in the literature. We included observational studies, retrospective studies, case series, survey-based studies, and review articles but excluded letters. Two of the authors (C.-H.H. and N.-C.H.) independently screened the searched publications to exclude duplicates. Only publications in English were included. No study was excluded according to the type of study or country.

Publications were selected from countries all over the world, and their important findings and conclusions were extracted from multiple aspects, providing key information and an overall understanding. However, many results may not be suitable to compare directly because different countries had different infection situations at different times, together with different infection control measures and responses, and different healthcare systems. Therefore, the results were summarized in an organized and focused manner. Unfair rankings or deliberate comparisons of advantages and disadvantages should be avoided among countries. Only a few comparable data, including (1) reduction percentage of all surgeries or emergency surgery volume, (2) maximal reduction percentage of elective surgery, (3) reduction percentage of emergency or outpatient visits, and (4) reduction percentage of orthopedic cases or referrals, were compared as much as possible.

## 3. Results

**Figure 1:**
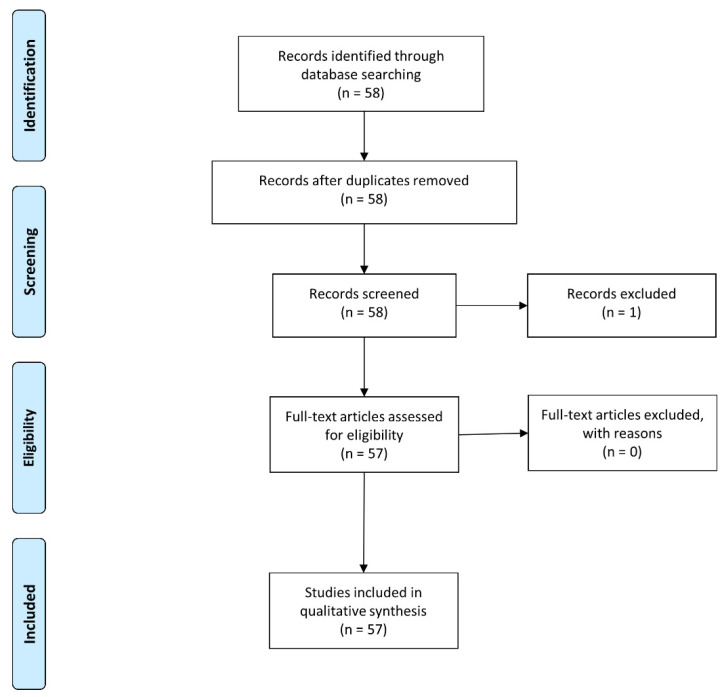
Literature selection process according to the Preferred Reporting Items for Systematic Reviews and Meta-Analyses guidelines.

The systematic search titles using the following items: ‘COVID-19’ AND ‘impact’ AND ‘orthopedic’ yielded 20 articles, while that using the following items: ‘COVID-19′ AND ‘impact’ AND ‘orthopaedic’ yielded 38 articles. In total, 58 studies were identified. We excluded one article because it was a letter to the editor (level of evidence: five). No prospective studies were found in this literature search. All included studies had a level of evidence of three or four. Finally, 57 articles were found to be eligible for further review and analysis according to the selection criteria. Most studies were conducted in Europe (n = 26), followed by Asia (n = 14) and North America (n = 9). Besides, one study was conducted in South America, one in the Middle East, one in Africa, and one in Australia. The European studies were conducted in the United Kingdom (n = 12), Italy (n = 5), Germany (n = 2), France (n = 1), Portugal (n = 1), Spain (n = 1), Greece (n = 1), Switzerland (n = 1), and Ireland (n = 1). The Asian studies were conducted in India (n = 5), Hong Kong (n = 2), Taiwan (n = 2), China (n = 1), Malaysia (n = 1), Singapore (n = 1), and South Korea (n = 1). In addition, the North American studies were conducted in the United States of America (n = 8) and Canada (n = 1). The literature selection was performed in accordance with the PRISMA guidelines [2], and the flow diagram is shown in Figure 1. Table 1 describes the detailed characteristics of all the included studies.

The impact of the pandemic on orthopedics was subdivided into orthopedic training and application, performance, work loading, change of practice, research work, and other psychological factors. The interesting results of each peer-reviewed publication were selected and reviewed. The remaining few studies that focused on specific orthopedic analysis metrics that were too trivial to be discussed in this review.

### 3.1. Impact on Orthopedic Training and Application

A total of 12 of the 57 included studies reported an impact on orthopedic training and application. Most studies were conducted in the United States of America (n = 6, 40%), followed by Europe (n = 4); this may show that the COVID-19 has influenced their training and application process to a greater extent. In most countries, training and application are still affected or even suspended. This may stem from lockdowns or restrictions and preventive measures, such as social distancing.

#### 3.1.1. Impact on Training

More studies (n = 8) focused on the impact of the pandemic on the training process (Table 2). A total of three perspective/narrative studies conducted in the United States of America (n = 2) and the United Kingdom (n = 1) described reduced surgical exposure of trainees and cancellation of examinations and courses that differed in training years [6,11]. Alternative supplementary learning methods were recommended [24].

There were three studies [7,26,29] that were questionnaire surveys conducted in Ireland, India, and South Korea. Sheridan et al. found that the average total number of surgeries per trainee was found to be 40.6 in 2019, which significantly dropped to 18.3 during the pandemic in 2020. Moreover, three trainees (7.69%) were infected with COVID-19 [26]. Upadhyaya et al. revealed that 65.1% of postgraduate students indicated that there were no clinical courses. 71.6% had problems completing their thesis. About 94% stated that their surgical and clinical training was affected [29]. The survey conducted among orthopedic residents by Chang et al. revealed a significant decrease in the average working time, lecture education hours, and discussion time for clinical cases (*p* < 0.001). In contrast, the use of virtual teaching methods increased significantly (*p* < 0.001). However, satisfaction with virtual teaching methods was significantly lower than that with traditional teaching methods [7].

Two large-scale questionnaire surveys [14,19] in multiple nations provide more comprehensive data, and the similar 20.9% and 23.1% redeployment rates of trainees was noteworthy. The survey conducted by Gonzi et al., in four nations revealed that 23.1% of trainees were reassigned to positions not related to surgery. Further, 42.9% did not receive clinic training in fractures as planned, and 63.8% did not gain sufficient experience in their affiliated subspecialties and preferred repeat training [14]. Another survey conducted by Megaloikonomos et al., in 23 European countries revealed that 20.9% of trainees were redistributed to COVID-19 units. 52.1% participants said that teacher-led teaching was limited; 46.3% were forced to change to self-learning; and surgical training was severely hindered in 58.6% of trainees. Meanwhile, 58.2% expressed concerns about not being able to meet their annual training goals, while one in four hoped to have one more year of training [19].

#### 3.1.2. Impact on Application Process

A total of four studies [3,10,49,54] conducted in the United States of America (n = 3) and Canada (n = 1) focused on the application process (Table 3). There were three perspective/narrative studies [3,49,54] that discussed the impact of COVID-19 on the application process and offered potential strategies. In response to the impact on matching, there is a strong need for a thorough understanding of the drastic adjustment in the process [3]. Adaptation to virtual interviews was proposed if it should become the new standard [49], and online and social media tools should be adopted to promote programmes [54]. Questionnaire surveys of medical students in the United States of America revealed suprising differences by gender and race. Significantly more women than men said they were ‘unlikely’ to apply for orthopedic residency (14.9% vs. 5.5%, *p* < 0.001). There were significantly more African American students (16.9%) to report ‘unlikely’ to apply than non-Hispanic American students (8.8%) (*p* < 0.001). A total of 88.9% of students also stated that they had ‘much less’ or ‘slightly less’ chances to participate in full training of surgery to get appropriate choices for future application [10].

### 3.2. Impact on Global Orthopedic Performance

A total of 15 of the 57 included studies focused mainly on the impact of the pandemic on the clinical performance volume. Most studies were conducted in Europe (n = 9), followed by Asia (n = 3), North and South America (n = 2), and Australia (n = 1). From these major publications from various countries, we can understand the real impact on orthopedic performance globally. In general, the performance volumes all inevitably declined. Even in some countries without lockdowns or restrictions, there was still a slight decrease, which may have been additionally affected by psychological fear. We believe that valuable experiences can be gained from these important research results in various countries.

#### 3.2.1. Europe

A total of nine studies [5,12,13,15,17,41,45,53,59] that reported the global service impact of the pandemic were conducted in Italy (n = 3), the United Kingdom (n = 2), Germany (n = 2), Portugal (n = 1), and Greece (n = 1) (Table 4).

An Italian study during lockdown showed that the mean age of the COVID-19 group (51.9 years) was significantly higher than that of the 2019 group (*p* < 0.0001) [5]. Another Italian study had unique results. During the lockdown period, urgent surgical activities for spinal diseases have increased with a low rate of COVID-19 infection (3.9%) [13]. Another Italian study revealed a decrease (−18.0%) in emergency room visits. Emergency room deaths increased by 220%. Orthopedic pathway rates decreased by −26.8%, while trauma rates at home increased by +19.1% [17].

A national survey in Germany revealed that significant financial and personnel changes had occurred, resulting in an average reduction of 49.4% in operating room capacity and an estimated 29.3% loss in revenue. In addition, 14.7% of physicians were reassigned [15]. Another German study focused on the emergency department during a lockdown for 35 days. The total number of orthopedic trauma patients (lockdown vs. control, 30.91 vs. 52.06, respectively) and daily number of patients (lockdown vs. control, 106.94 vs. 167.54, respectively) decreased as the incidence of domestic violence, home injuries, bicycle accidents, and drug abuse increased [45].

A United Kingdom study during lockdown revealed that emergency visits of orthopedic patients dropped to 58.6%. The number of orthopedic visits yielded a reduction rate of 57.4% [12]. A United Kingdom orthopedic team had created a one-week ‘one-stop’ clinic for ambulatory patients with minor injuries to reduce the pressure on the emergency room. About 700 patients who should have been treated in the emergency room were moved to the minor injury unit. The clinic had only 2% (15 patients) revisit rate, of which only four patient needed further management [41].

#### 3.2.2. Asia

Three studies [18,31,56] reported the impact on orthopedic performance in Asia (Table 5). A multicenter study in India showed a significant reduction of 1266 total trauma cases during the lockdown period (62.7% reduction rate, *p* < 0.01). The leading causes of trauma were road traffic accidents, with a 77.9% reduction rate (n = 1343 vs. n = 298) during the lockdown [18].

A Hong Kong study showed that orthopedic surgery performance dropped significantly by 44.2%, and the elective to emergency ratio of the surgery decreased to 1:3.78. The number of inpatients and outpatients dropped significantly by 41.2% and 29.4%, respectively. The surgical treatment rates for upper and lower extremity fractures dropped significantly by 23% and 20%, respectively, and the rates of elective ligament reconstruction and joint replacement dropped significantly by 74% to 84% [31].

A study conducted in Taiwan revealed a 22–37% reduction in the number of inpatients, 20–29% reduction in the number of outpatients, and 18–35% reduction in the number of orthopedic surgeries during the COVID-19 pandemic [56].

#### 3.2.3. America

Two studies [33,40] reported the impact on orthopedic performance in America (Table 6). A study in the United States included 2830 cases for multi-subspecialty percentages analysis (pre-COVID-19 vs. post-COVID-19: 1917 vs. 913). A significant increase in hip surgery (+3.5%) and a significant decrease in wrist and hand surgery (−2.6% and −2.1%) were found [40]. A study in Chile showed that a 22.8% drop in orthopedic surgery performance. All types of surgical performance were affected, with knee arthroplasty having the greatest impact (−64%), followed by knee ligament reconstruction (−44%) and hip replacement (−41%). Trauma surgery/fracture was least affected [33].

#### 3.2.4. Australia

An Australian study (Table 7) showed a 15.6% decrease in the total number of emergency surgeries and a 30.8% decrease in orthopedic hospital admissions compared to the same period in 2019. Accidents caused by bicycles increased significantly to 11% of all accidents. During the pandemic, the number of multiple injuries, sports injuries, and work injuries decreased [50].

#### 3.2.5. Comparison of the Reduction Percentage in Various Countries

**Figure 2:**
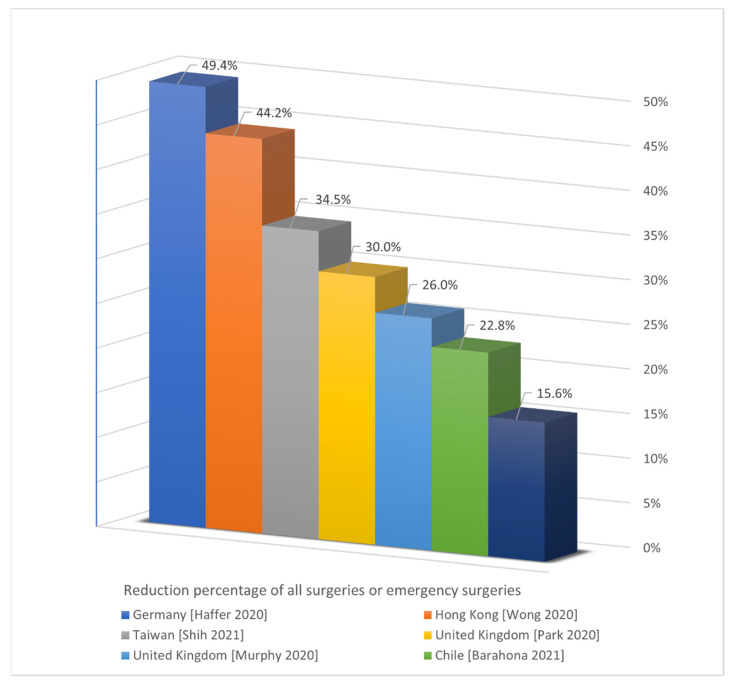
Reduction percentage of all surgeries or emergency surgeries [15,20,22,31,33,50,56].

Percentage reductions in different countries may probably be the few metrics that can be compared. However, it is not possible to obtain all relevant information from every country. Studies conducted in some countries may not include these data. First, the percentages of volume reduction reported in the included studies for all surgeries or emergency surgeries, in descending order, were 49.4% (mean of estimation from 43 respondents) in Germany [15], 44.2% (795 ± 115.1/443.6 ± 25.8 weekly operations) in Hong Kong [31], 34.5% (47.0 ± 8.4/30.8 ± 5.4 weekly surgery) in Taiwan [56], 30% (90/63 total operations in first month) and 26% (53/39 weekly operations) in the United Kingdom [20,22], 22.8% (128,735/99,333 surgery in a country) in Chile [33], and 15.6% (173/146 emergency operations) in Australia [50] (Figure 2). Interestingly, even though Hong Kong and Taiwan avoided lockdowns or restrictions and got very few COVID-19 cases during the first wave, they still had a significant impact on surgeries. This may be a psychological factor due to the proximity to China and the large flow of people among countries.

**Figure 3:**
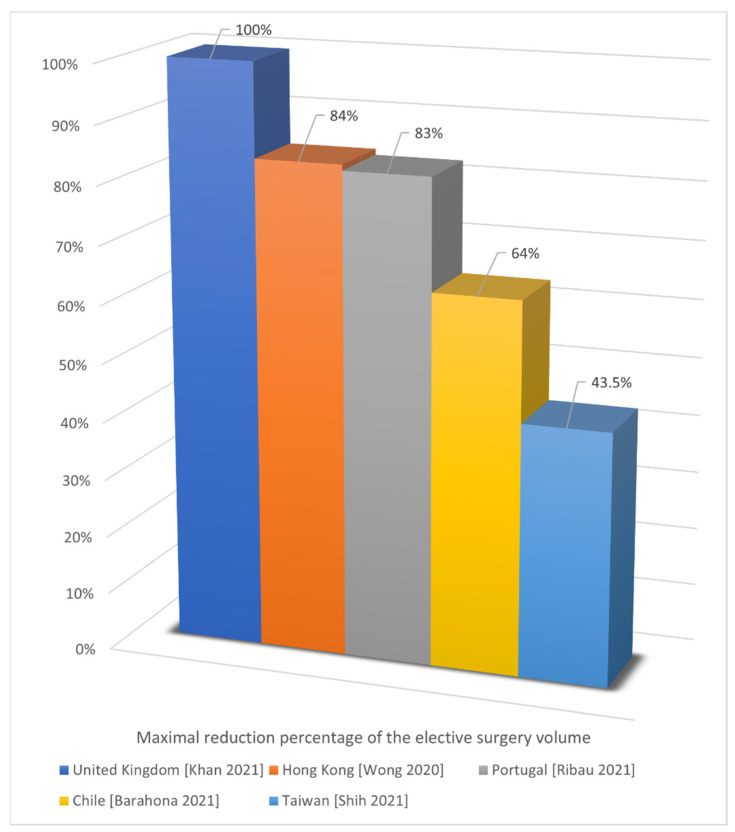
Maximal reduction in elective surgery performance [31,33,43,53,56].

Second, the maximal percentage reductions in elective surgery performance reported in the included studies, in descending order, were 100% (91% of respondents reported all elective operating had been cancelled) in the United Kingdom [43], 84% (14.9 ± 4.6/2.4 ± 2.2 weekly elective anterior cruciate ligament reconstruction surgery) in Hong Kong [31], 83% (587/100 elective surgery) in Portugal [53], 64% (28.23/10.13 per 100,000 inhabitants, maximum in total knee arthroplasty) in Chile [33], and 43.5% (41.3 ± 8.1/22.8 ± 3.3 weekly elective surgery) in Taiwan [56] (Figure 3). It could be noted that the maximal percentage reductions were more than 50% in most countries. The relatively small percentage reduction in Taiwan may be due to escaping lockdowns and restrictions, that is, most elective surgeries could still be scheduled as normal [60]. Taiwan successfully stopped COVID-19 spread without implementing any lockdown in the first wave and had effectively adopted many preventive strategies, including mandating the use of face masks in public [61].

**Figure 4:**
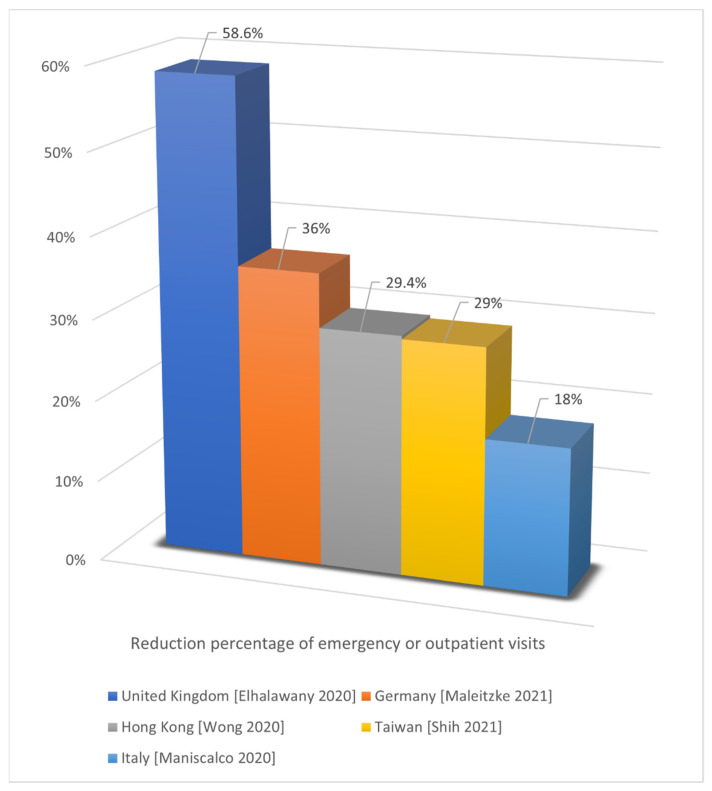
Reduction in emergency or outpatient visits [12,17,31,45,56].

**Figure 5:**
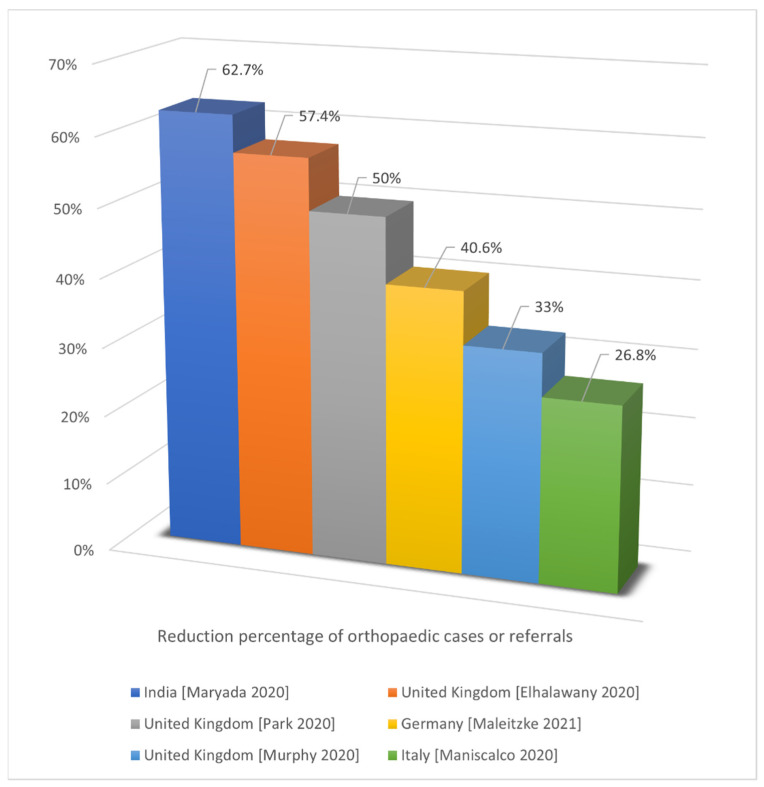
Reduction in orthopedic cases or referral [12,17,18,20,22,45].

Third, the percentage reductions in the emergency or outpatient visits reported in the included studies were 58.6% (4777/1978, emergency visits) in the United Kingdom [12], 36% (167.54/106.94 daily emergency trauma visits) in Germany [45], 29.4% (11,693 ± 2240/8261 ± 1104 weekly outpatient visits) in Hong Kong [31], 29% (5100/3621 monthly outpatient visits) in Taiwan [56], and 18% (32,980/27,042 emergency visits) in Italy [17] (Figure 4). The reduction percentages of orthopedic cases or referrals reported in the included studies were 62.7% (2020/754 cases) in India [18], 57.4% (1729/736 orthopedic presentations), 46.3% (162/87 acute trauma referrals), and 33% (112/75 weekly referrals) in the United Kingdom [12,20,22], 40.6% (52.06/30.91 daily orthopedic trauma cases) in Germany [45], and 26.8% (4007/2934 orthopedic pathways) in Italy [17] (Figure 5). The data from the study conducted in Italy only focused on emergency room visits and showed a relatively smaller reduction percentage; those from some other studies focused on outpatient visits. 

Due to differences in infection status, early response, infection control measures and administrative strategies (restrictions or lockdowns) across countries, the comparison of the percentage reductions across countries may only reflect part of the actual situation and provide a general understanding. Moreover, although these reductions were all caused by the first wave of the pandemic, they were not observed at the same time. 

### 3.3. Impact on Work Loading

Relative to the performance of the entire hospital or department, the work loading involves the individual itself. Three United Kingdom studies [20,22,27] focused on the impact of the pandemic on an individual’s burden (Table 8). One study showed a significant decrease in the average number of referrals per week (−33%) and the number of surgeries per week (−26%). The number of referrals for soft tissue injuries, wounds, natural joint dislocations, and simple fractures significantly decreased. The number of referrals related to specific injuries, such as domestic abuse, non-accidental injury, hip fracture, prosthetic joint dislocation, and periprosthetic fracture, did not change [20]. Another study revealed that the number of referrals of acute trauma decreased by nearly 50%, similar for both children and adults; meanwhile, the number of patients requiring hospitalization increased significantly by 19%. During the pandemic, the total number of surgeries decreased by 30%, with 14% reduction in the use of anesthesia techniques that generate aerosols [22]. A study of pediatric trauma during lockdown showed significantly fewer patients receiving counseling and face-to-face follow-up, and a 68% reduction in the number of acute pediatric trauma referrals [27].

### 3.4. Change of Practice

Four studies [28,36,42,43] conducted in Malaysia, Spain, India, and the United Kingdom and one large questionnaire survey conducted in 45 countries [37] focused on the change of practice (Table 9).

A Malaysian national survey showed that the majority of respondents continued to work (94.9%), operate outpatient clinics (75.3%), and perform emergency (95.5%) and semi-emergency surgeries (85.2%). Among surgeons, 61.9% suffered income losses, and 84.8% had adopted more conservative management strategies due to COVID-19 [28]. A Spanish questionnaire showed that 85.7% of orthopedic surgeons were forced to decrease their surgical practice by 50–100%. A total of 52% revised the indications for the treatment of various fractures, with differences between community hospital and medical center. About 46% were asked to work with staff from other units or departments, and 43% felt that their jobs were underutilized [36]. In an Indian nationwide questionnaire, there was a significant change of practice in individual hospital protocols (91.7%). The majority of patients (88%) found that both trauma and non-traumatic surgery were seriously affected by more than half. Most surgeons (90%) did not upgrade or improve the current equipment of the operating room [42]. Another nationwide survey in United Kingdom showed that all respondents (n = 202) stated that their daily practice was interrupted. Approximately 91% stated that all elective surgeries had been cancelled. A total of 70% stated interruption of trauma surgery. Only 24% reported that trauma surgery was performed as usual. Approximately 55% reported that the operation of their elective surgical clinics was completely cancelled; meanwhile, 38% of respondents stated that their elective surgery clinic was operating at reduced capacity, and non-urgent appointments were postponed. There were 69% that had reduced practice, and only 9% of fracture clinics operated normally. Approximately 67% of clinicians reported cancellation of teaching and study leave [43].

Finally, a larger questionnaire survey was conducted among orthopedic surgeons in 45 countries. During the survey period, 79% of respondents reported a lockdown in their areas, resulting in a change of personal practice. The average weekly number of outpatient appointments fell from 67.89 to 11.79 during the pandemic. The average weekly number of surgeries has decreased, from 6.89 to 1.25 during the pandemic [37]. Additionally, previous research has shown that patients with COVID-19 who are undergoing surgery have a significantly higher risk of postoperative complications and an increased risk of mortality [62]. Therefore, appropriate changes in practice may be beneficial in the COVID-19 era.

### 3.5. Psychological Impact

Psychological factors also played a role. Fear or stress may stem from drastic environmental changes. In the early days of the pandemic, rumors, false news or exaggerations in the mass media, and lack of personal protective equipment may have caused widespread panic among the public. There were two studies [25,32] focused on the psychological impact of the pandemic. A questionnaire survey conducted in India showed that 40.5% of orthopedic surgeons reported that they had mild pressure, and 22.5% reported that they must be under stress. The percentage of orthopedic surgeons feeling ‘a lot of stress’ had increased with declining age. Uncertainty of returning to work and disruption of life–work balance were major factors strongly associated with ‘absolutely stressed’ status [25]. Another Singapore survey revealed that 51.6% had ≥7 positive responses. ‘restrictions’ (72.6%), ‘changes in personal plans’ (72.6%), and ‘Work adjustments’ (74.2%) yielded the most positive responses. Meanwhile, the least positive responses (21.0%) were ‘financial issues’ [32].

### 3.6. Impact on Orthopedic Research Work

Only one included study discussed the impact of the pandemic on research work. A questionnaire survey was conducted in 45 countries. Among orthopedic surgeons, 82.8% reported research activities continued during the pandemic, with the majority reporting the recruitment of participant stopped (64.15%) or decreased (29.9%) [37]. Another editorial comment that specifically focused on the impact of COVID-19 on research reported that most research laboratories have been closed due to redeployment of the staff to help conduct COVID-19 trials [63].

### 3.7. Implications of Telemedicine

The COVID-19 pandemic has forced a radical and rapid redesign of the way healthcare systems are delivered. One of the most notable ongoing changes is the unprecedented acceleration in the expansion of telemedicine. The pandemic has encouraged the realization of virtual teaching, virtual training, and virtual consultation. Many included studies discussed the implications of telemedicine.

Sugand et al. showed that outpatient telemedicine and virtual fracture clinics were used significantly more, and significantly fewer patients had face-to-face consultation [27]. A nationwide questionnaire survey conducted in Malaysia revealed that approximately 19.3% of surgeons started using telemedicine facilities [28]. A larger questionnaire survey among orthopedic surgeons was conducted in 45 countries. 39.4% of respondents started to use virtual appointments of outpatients for the first time [37]. The survey by Chang et al. revealed the implementation of virtual teaching increased significantly among orthopedic residents (*p* < 0.001). However, satisfaction with traditional teaching methods was significantly higher than that with virtual teaching methods [7].

## 4. Discussion

Since the global spread of COVID-19 in early 2020, its current influence on daily life has weakened, but has continued for a longer time than what most people expected. The purpose of this study was to analyze the current literature on the impact of the COVID-19 pandemic on the overall field of orthopedics, including orthopedic training and application, performance, work loading, change of practice, research work, and other psychological factors.

The included studies showed a dramatic decline in nearly all aspects of orthopedics. The total number of surgeries or emergency surgeries decreased by up to 49.4% [15], and the total number of elective surgeries decreased by up to 100% [43]. Many countries worldwide have imposed many restrictions or strategies to block the spread of infection and prevent the healthcare system from shutting down. Furthermore, people were asked or forced to stay at home. Consequently, the number of motor vehicle accidents has decreased in some countries [18,52]. For example, Maryada et al., reported a 77.9% reduction in the number of road traffic accidents during lockdown at eight teaching hospitals in India [18]. However, the number of cycling-related accidents increased significantly in Australia during the pandemic [50]. Studies on accidents at home have yielded different results. Oguzkaya et al., reported that the proportion of domestic accidents was as high as 48.5% for all injury mechanisms; this proportion significantly increased during the pandemic [47]. It is reasonable that owing to government regulations, people will spend more time at home. They will also avoid going to the hospital for fear of possible infection with COVID-19. Although there are undoubtedly some injuries requiring urgent treatment, telemedicine or virtual consultation may be a good alternative option to provide rapid and safe healthcare services in some countries [24,27,28,30,35,48]. Teo et al. reported that 19.3% of orthopedic surgeons started to use telemedicine owing to COVID-19 in Malaysia [28]. However, further rigorous studies are necessary to evaluate the outcomes of patients using telemedicine or virtual consultations. For orthopedic surgeons, several congresses and courses (e.g., EFORT, AOTrauma and similar) or annual meetings (e.g., AAOS, ORS) were cancelled or “virtualized”. Recently, more and more other studies discussed the implications of telemedicine in orthopedics from various aspects. [64,65,66,67,68,69,70,71,72,73,74,75,76,77].

Is there a possibility that fear of the pandemic has led to a situation in which emergency patients opt to visit a family physician? We believe that this generally occurs; however, it is difficult to obtain data in these situations, and therefore, the included studies fail to present such data. Nevertheless, it can be observed from some studies that the reduction in outpatient volume is less than that of the emergency department. However, other studies have reported conflicting results because of outpatient clinics being forced to shut down in order to maintain the emergency department capacity, and thus leading to inconsistent results.

The total number of surgeries in most hospitals has dropped significantly, which is reasonable and unsurprising. There are multiple reasons for this result, including the decrease in the number of emergency visits, cancellation of elective surgery, and psychological fear of patients. As the only exception, Ghermandi et al. showed that the surgical activities of Italian oncology and spine surgery have increased. This may be attributed to the combined neurological or functional deficits in these diseases that cannot be delayed during the treatment process [13]. Therefore, preventive strategies should be in place to allow the patients to undergo timely orthopedic surgeries, even as the pandemic persists. At the same time, we should keep the employees safe with appropriate protection. Developing an appropriate surgical scheduling algorithm for orthopedic patients may achieve this goal.

It would be interesting to compare the impact on elective surgery (e.g., arthroplasty) in countries with no lockdown (Sweden) or limited lockdown (New Zealand). There was a 54% drop in the rate of elective joint replacement surgery in Sweden in April 2020 [78]. However, the New Zealand Government committed to an elimination strategy with a level four alert, declaring a state of national emergency on 25 March 2020. Level four was the most stringent and included the complete cessation of elective surgeries [79]. As a result, there was a 100% drop in elective surgeries. Therefore, the political measure of lockdown is a key factor in the reduction in elective surgery.

Additionally, it is noteworthy that in some countries there was a severe shortage of ventilators and personal protective equipment (especially early in the pandemic), which could have exacerbated the “covid” effect in the early stages of the pandemic. Conversely, vaccination programs introduced later in the pandemic were more likely to “encourage” surgeons to perform elective procedures.

What else can we learn from current impact to cope with possible recurrence of the pandemic in the future? We believe that it is essential for physicians to set up urgent measures in ordinary circumstances, including the second-level/alternative duty roster for the period of the pandemic or a special duty roster for the period of lockdown under the principle of staff grouping and workplace partition. It is also essential to maintain flexible allocation of manpower and plan the reserve of support manpower to cope with emergency staff shortages owing to redeployment.

This study had some limitations. Most importantly, specific search keyword restrictions on the inclusion criteria might have resulted in excluding studies that discussed the impact of COVID-19 on orthopedics but did not use the relevant keyword in the title. In addition, the exclusion criteria were minimized, and the level of evidence of the included studies was relatively low (three or four). It was also difficult to compare among the various countries owing to the differences in their healthcare systems, infection control measures and responses, and infection situations at different times. Moreover, most studies specifically mentioned only the first wave of the COVID-19 pandemic. Therefore, compared with that of the current situation, the impact seems to have been overestimated. Another limitation of the present study was that the psychological aspect of “risking life and going to a hospital” for an elective surgery was not considered in the included cross-sectional studies. This could be a key factor limiting elective surgeries early in the pandemic, especially in the elderly population. However, in the later stages, due to vaccination programs, a higher percentage of patients would be willing to undergo such procedures.

Finally, a serious limitation may occur in the oversimplification of lockdown classification. We distinguished the status of the lockdown based on the time points covered by each study and the conditions described in its content. Several countries had adopted various strategies which were constantly modified over time. For example, Germany implemented different rules during various waves. According to the situation in 2020, it could be roughly divided into three phases: (1) the first wave in March and April (the first lockdown began on 16 March with school closures and prohibited visits to nursing homes, and one week later, many public places were shut down as well, including restaurants, most retail stores, hotels, bars, museums, libraries, theaters, cinemas, and playgrounds); (2) a relaxation phase during summer with gradual relaxation of the lockdown measures; and (3) a second wave starting in October with partial lockdown or “lockdown light” announced on 28 October. In contrast to the first lockdown, retail stores and schools remained open [80]. Conversely, the main advantage of this systematic review of the literature is the heterogeneous source of the included studies, which provides a good overall view of the global impact of the COVID-19 pandemic on the field of orthopedics.

## 5. Conclusions

Although orthopedic surgeons do not seem to be on the frontline fighting against the pandemic, the field of orthopedics is obviously affected. Most studies have reported that the number of cases in all aspects decreased significantly. Orthopedic education and training, research, and psychological pressure, which have been less noted, have also been significantly impacted. Externally, the overall change seems to be rooted in patients’ fear, lockdowns, and restrictions. Internally, the overall change seems to be attributable to the redeployment or redistribution of personnel in response to the pandemic. In the future, regardless of whether the pandemic has not stopped, it will be important to maintain the normal operation of treatment and surgery to avoid sequelae caused by delayed treatment. It is important for orthopedic surgeons to prepare more sufficient, flexible, and reservable staffing measures, proper preventive strategies, and surgical scheduling algorithms and set up dedicated venues and equipment for routine telemedicine with staff training for virtual teaching or consultations in cases of future impacts on orthopedics. 

## Figures and Tables

**Table 1:** Characteristics of relevant publications.

No	Study	Year	Country	Region	Restriction * (Lockdown)	Study Method	Focus
1	Aiyer et al. [3]	2020	USA	North America	Partial	Narrative/Perspective	Resident Application
2	Alyami et al. [4]	2020	Saudi Arabia	Middle East	Complete	Narrative/Perspective	Performance/Training
3	Andreozzi et al. [5]	2020	Italy	Europe	Complete	Retrospective	Performance/Orthopedic Trauma
4	Bernstein et al. [6]	2020	USA	North America	Partial	Perspective/Reflection	Resident and intern training
5	Chang et al. [7]	2020	South Korea	Asia	Partial	Survey/Questionnaire	Training/Residency
6	Clement et al. [8]	2020	UK	Europe	Partial	Multicenter, retrospective	Surgical Risk Assessment
7	Costa et al. [9]	2020	Italy	Europe	Partial	Retrospective	Prevention measures
8	Danford, et al. [10]	2020	USA	North America	Partial	Survey/Questionnaire	Resident Application
9	Dattani et al. [11]	2020	UK	Europe	Partial	Perspective/Narrative	Training/Trainees
10	Elhalawany et al. [12]	2020	UK	Europe	Complete	Retrospective	Performance/Orthopedic emergency
11	Ghermandi et al. [13]	2020	Italy	Europe	Partial	Retrospective	Performance/Orthopedic oncology and spine
12	Gonzi et al. [14]	2020	UK	Europe	Partial	Survey/Four-nation questionnaire	Training/Trainees
13	Haffer et al. [15]	2020	Germany	Europe	Partial	Nationwide questionnaire survey	Performance/Orthopedic and Trauma Surgery
14	Mackay et al. [16]	2020	UK	Europe	Partial	Retrospective cohort	Surgical Risk Assessment
15	Maniscalco et al. [17]	2020	Italy	Europe	Complete	Retrospective	Performance/Orthopedics and Emergency Room
16	Maryada et al. [18]	2020	India	Asia	Complete	Multi-center retrospective	Performance/Orthopedic Trauma
17	Megaloikonomos et al. [19]	2020	Europe	Europe	Partial	23 European countries questionnaire	Training/Trainees
18	Murphy et al. [20]	2020	UK	Europe	Partial	Retrospective	Work loading/Orthopedic
19	Ong et al. [21]	2020	Hong Kong	Asia	Partial	Narrative/Perspective	Performance/Education/Research
20	Park et al. [22]	2020	UK	Europe	Complete	Retrospective	Work loading/Orthopedic trauma
21	Phillips et al. [23]	2020	N/A	N/A	Complete	Review	Orthopedic care
22	Richardson et al. [24]	2020	USA	North America	Partial	Perspectives	Training/medical student
23	Sahu et al. [25]	2020	India	Asia	Complete	Questionnaire survey	Psychological/orthopedic surgeon
24	Sheridan et al. [26]	2020	Ireland	Europe	Partial	Questionnaire	Training/Trainees
25	Sugand et al. [27]	2020	UK	Europe	Complete	Multi-center retrospective	Work loading/Pediatric orthopedic trauma
26	Teo et al. [28]	2020	Malaysia	Asia	Partial	Nationwide questionnaire survey	Practice Change/Surgeon
27	Upadhyaya et al. [29]	2020	India	Asia	Partial	Questionnaire survey	Training/Trainees
28	Wallace et al. [30]	2020	UK	Europe	Complete	Perspectives	Orthopedic surgery and trauma
29	Wong et al. [31]	2020	Hong Kong	Asia	Partial	Retrospective cohort	Performance/Orthopedic and Trauma
30	Wong et al. [32]	2020	Singapore	Asia	Partial	Questionnaire survey	Psychological/orthopedic outpatient setting
31	Barahona et al. [33]	2021	Chile	South America	Partial	Retrospective	Performance/Orthopedic surgery
32	Blum et al. [34]	2021	N/A	N/A	N/A	Review	Performance/Orthopedic and Trauma Surgery
33	Chatterji et al. [35]	2021	N/A	N/A	N/A	Rapid Review	Miscellaneous
34	Garcia et al. [36]	2021	Spain	Europe	Partial	Questionnaire survey	Change of practice/Orthopedic Surgeon
35	Gibbard et al. [37]	2021	N/A	N/A	Partial	Global (45 countries) questionnaire survey	Change of practice/Pediatric Orthopedic Surgeon
36	Giordano et al. [38]	2021	N/A	N/A	Partial	14 Latin American countries questionnaire survey	Financial, Psychosocial/Orthopedic Trauma surgeon
37	Green et al. [39]	2021	UK	Europe	Partial	Retrospective cohort	Length of stay/total joint arthroplasty
38	Heaps et al. [40]	2021	USA	North America	Partial	Retrospective cohort	Performance/multi-subspecialty
39	Howles et al. [41]	2021	UK	Europe	Partial	Retrospective cohort	Performance/Minor injury one-stop unit
40	Jain et al. [42]	2021	India	Asia	Complete	Nationwide questionnaire	Change of practice/Orthopedic Surgeon
41	Khan et al. [43]	2021	UK	Europe	Partial	Nationwide questionnaire	Change of practice/Orthopedic Surgeon
42	Ma et al. [44]	2021	Taiwan	Asia	No	Retrospective cohort	Screening/Emergency room/Trauma
43	Maleitzke et al. [45]	2021	Germany	Europe	Complete	Retrospective cohort	Performance/Orthopedic trauma
44	Moretti et al. [46]	2021	Italy	Europe	Complete	Nationwide Questionnaire	Psychological/Gender-specific
45	Oguzkaya et al. [47]	2021	Turkey	Asia and Europe	Partial	Multi-center retrospective	Orthopedic fracture characteristics
46	Paul et al. [48]	2021	USA	North America	Partial	Nationwide Questionnaire	Change of practice/Elective procedures/Telehealth and income
47	Peebles et al. [49]	2021	USA	North America	Partial	Narrative Review/Perspective	Sports Fellowship Application
48	Probert et al. [50]	2021	Australia	Australia	Complete	Retrospective	Performance/Orthopedic trauma
49	Qian et al. [51]	2021	China	Asia	Partial	Retrospective	Performance/orthopedic trauma
50	Rachuene et al. [52]	2021	South Africa	Africa	Complete	Multicenter retrospective	Performance/orthopedic trauma
51	Ribau et al. [53]	2021	Portugal	Europe	Complete	Retrospective	Performance/orthopedic trauma
52	Shah et al. [54]	2021	Canada	North America	partial	Narrative Review/Perspectives	Residency application
53	Sharma et al. [55]	2021	India	Asia	Complete	Questionnaire	Psychological/Change of practice
54	Shih et al. [56]	2021	Taiwan	Asia	No	Retrospective	Psychological/Performance
55	Unterfrauner et al. [57]	2021	Switzerland	Europe	Complete	Retrospective	Surgical site infections/Complications
56	Van Heest et al. [58]	2021	USA	North America	Partial	Symposium summary/Review	Training/Orthopedic Graduate Medical Education
57	Vasiliadis et al. [59]	2021	Greece	Europe	Partial	Retrospective cohort	Performance

* When the research content clearly indicated that there was a lockdown or the research period was during the lockdown, it was considered ‘complete’. Conversely, when the study clearly stated that there was no lockdown at all, it was considered ‘none’. When it was not specified, it was designated as ‘partial’. The indication of this status is for simple differentiation only and does not absolutely reflect the actual situation.

**Table 2:** Characteristics of relevant publications on orthopedic training.

Orthopedic Training				
Study	Country	Method of Questioning	Subject	Number of Respondents
Bernstein et al. [6]	USA	Perspective/Reflection	Resident and intern	N/A
Chang et al. [7]	South Korea	Web-based survey questionnaire	Resident	229
Dattani et al. [11]	UK	Perspective/Narrative	Trainees	N/A
Gonzi et al. [14]	UK	Survey/Four-nation questionnaire	Trainees	101
Megaloikonomos et al. [19]	Europe	23 European countries questionnaire	Trainees	327
Richardson et al. [24]	USA	Perspectives	medical student	N/A
Sheridan et al. [26]	Ireland	Questionnaire	Trainees	40
Upadhyaya et al. [29]	India	Questionnaire survey	Post-graduate trainees	138

**Table 3:** Characteristics of relevant publications on orthopedic application.

Orthopedic Application				
Study	Country	Method of Questioning	Subject	Number of Respondents
Aiyer et al. [3]	USA	Narrative/Perspective	Residency	N/A
Danford, et al. [10]	USA	Survey/Questionnaire	Residency	462
Peebles et al. [49]	USA	Narrative Review/Perspective	Sports Fellowship	N/A
Shah et al. [54]	Canada	Narrative Review/Perspectives	Residency	N/A

**Table 4:** Characteristics of relevant publications on orthopedic performance in Europe.

Study	Country	Study Method	Focus	Settings Investigated	Number of Patients (% Change)
Andreozzi et al. [5]	Italy	Retrospective	Orthopedic trauma	trauma admissions	995/204 (−79%) [Age 41.4 ± 25.7/51.9 ± 24.8, *p* < 0.0001]
Elhalawany et al. [12]	UK	Retrospective	Orthopedic emergency	lockdown on orthopedic emergency presentations	4777/1978 (−58.6% emergency visits) 1729/736 (−57.4% orthopedic presentations)
Ghermandi et al. [13]	Italy	Retrospective	Orthopedic oncology and spine	Daily surgical activity	69/102 (+48%)
Haffer et al. [15]	Germany	Nationwide questionnaire survey	Orthopedic and trauma surgery	52 surgeons participated	Mean of estimation from 43 respondents (−49.4% operating room capacity)
Maniscalco et al. [17]	Italy	Retrospective	Orthopedics and emergency room	trend of emergency room accesses and events	32,980/27,042 (−18% emergency room accesses) 4007/2934 (−26.8% orthopedic pathways)
Howles et al. [41]	UK	Retrospective cohort	Minor injury one-stop unit	service provided to patients	700
Maleitzke et al. [45]	Germany	Retrospective cohort	Orthopedic trauma	trauma care in emergency departments	167.54/106.94 (−36% daily total cases) 52.06/30.91 (−40.6% daily orthopedic trauma cases)
Ribau et al. [53]	Portugal	Retrospective	Orthopedic trauma	lockdown period on the surgical activity	587/100 (−83% elective surgery)
Vasiliadis et al. [59]	Greece	Retrospective cohort	Orthopedic practice	everyday orthopedic practice	1042/550 (−47.2% emergency)

**Table 5:** Characteristics of relevant publications on orthopedic performance in Asia.

Study	Country	Study Method	Focus	Settings Investigated	Number of Patients (% Change)
Maryada et al. [18]	India	Multi-center retrospective	Orthopedic Trauma	lockdown on the trauma case load	2020/754 (−62.7% trauma) 1343/298 (−77.9% road traffic accidents)
Wong et al. [31]	Hong Kong	Retrospective cohort	Orthopedic and Trauma	All orthopedic practice	795 ± 115.1/443.6 ± 25.8 (−44.2% weekly operations) 14.9 ± 4.6/2.4 ± 2.2 (−84% weekly elective anterior cruciate ligament reconstruction) 11,693 ± 2240/8261 ± 1104 (−29.4% weekly outpatient visits)
Shih et al. [56]	Taiwan	Retrospective	Orthopedic practice	All orthopedic practice	47.0 ± 8.4/30.8 ± 5.4 (−34.5% weekly surgery) 41.3 ± 8.1/22.8 ± 3.3 (maximal −43.5% weekly elective surgery

**Table 6:** Characteristics of relevant publications on orthopedic performance in America.

Study	Country	Study Method	Focus	Settings Investigated	Number of Patients (% Change)
Barahona et al. [33]	Chile	Retrospective	Orthopedic surgery	Orthopedic surgery in a single country	128,735/99,333 (−22.8% surgery) 28.23/10.13 per 100,000 inhabitants (−64% maximum in total knee arthroplasty)
Heaps et al. [40]	USA	Retrospective cohort	multi-subspecialty	multi-subspecialty surgery percentages analysis	1917/913 (pre-COVID-19 vs. post-COVID-19)

**Table 7:** Characteristics of relevant publications on orthopedic performance in Australia.

Study	Country	Study Method	Focus	Settings Investigated	Number of Patients (% Change)
Probert et al. [50]	Australia	Retrospective	Orthopedic trauma	Lockdown on emergency orthopedic surgery	173/146 (−15.6% emergency operations)

**Table 8:** Characteristics of relevant publications on orthopedic work loading.

Study	Country	Study Method	Focus	Settings Investigated	Number of Patients (% Change)
Murphy et al. [20]	UK	Retrospective	Orthopedic trauma	trauma referrals	112/75 (−33% weekly referrals) 53/39 (−26% weekly operations)
Park et al. [22]	UK	Retrospective	Orthopedic trauma	trauma referrals and surgery for the first “golden” month	90/63 (−30% total operations in first month 162/87 (−46.3% acute trauma referrals)
Sugand et al. [27]	UK	Multi-center retrospective	Pediatric orthopedic trauma	Lockdown on acute pediatric orthopedic trauma referral caseload	302/97 (−68% acute pediatric trauma referrals)

**Table 9:** Characteristics of relevant publications on change of practice.

Study	Country	Study Method	Respondent	Major Change of Practice
Teo et al. [28]	Malaysia	Nationwide questionnaire survey	Orthopedic Surgeon	84.8% (189/223) make decision to manage more conservatively
Garcia et al. [36]	Spain	Questionnaire survey	Orthopedic Surgeon	52% modified the treatment indications
Gibbard et al. [37]	N/A	Global (45 countries) questionnaire survey	Pediatric Orthopedic Surgeon	79% (358/460) of respondents reported a lockdown, resulting in a change of practice
Jain et al. [42]	India	Nationwide questionnaire	Orthopedic Surgeon	91.7% (539/588) had significant changes made in individual hospital protocols
Khan et al. [43]	UK	Nationwide questionnaire	Orthopedic Surgeon	All 202 participants reported disruption to their daily practice 91% reported all elective operating had been cancelled

## Data Availability

The data presented in this study are available on request from the first author.
